# Structure and Thermomechanical Properties of Polyvinylidene Fluoride Film with Transparent Indium Tin Oxide Electrodes

**DOI:** 10.3390/polym15061483

**Published:** 2023-03-16

**Authors:** Vitaliy Solodilov, Valentin Kochervinskii, Alexey Osipkov, Mstislav Makeev, Aleksandr Maltsev, Gleb Yurkov, Boris Lokshin, Sergey Bedin, Maria Shapetina, Ilya Tretyakov, Tuyara Petrova

**Affiliations:** 1 Laboratory of Ferroelectric Polymers, Bauman Moscow State Technical University, 105005 Moscow, Russia; 2 Department of Electronics of Organic Materials and Nanostructures, N.M. Emanuel Institute of Biochemical Physics (IBCP), Russian Academy of Science (RAS), 119334 Moscow, Russia; 3 N.N. Semenov Federal Research Center of Chemical Physics, Russian Academy of Sciences, 119991 Moscow, Russia; 4 Department of Physical and Physico-Chemical Methods for Studying the Structure of Substances, A.N. Nesmeyanov Institute of Organoelement Compounds, Russian Academy of Sciences, 119334 Moscow, Russia; 5 Laboratory of Physics of Advanced Materials and Nanostructures, Moscow Pedagogical State University, 119991 Moscow, Russia

**Keywords:** PVDF, ITO, ferroelectric polymers, thermomechanical testing, structure, transparency, piezoelectric properties

## Abstract

This paper is devoted to the study of the structure and thermomechanical properties of PVDF-based ferroelectric polymer film. Transparent electrically conductive ITO coatings are applied to both sides of such a film. In this case, such material acquires additional functional properties due to piezoelectric and pyroelectric effects, forming, in fact, a full-fledged flexible transparent device, which, for example, will emit a sound when an acoustic signal is applied, and under various external influences can generate an electrical signal. The use of such structures is associated with the influence of various external influences on them: thermomechanical loads associated with mechanical deformations and temperature effects during operation, or when applying conductive layers to the film. The article presents structure investigation and its change during high-temperature annealing using IR spectroscopy and comparative results of testing a PVDF film before and after deposition of ITO layers for uniaxial stretching, its dynamic mechanical analysis, DSC, as well as measurements of the transparency and piezoelectric properties of such structure. It is shown that the temperature-time mode of deposition of ITO layers has little effect on the thermal and mechanical properties of PVDF films, taking into account their work in the elastic region, slightly reducing the piezoelectric properties. At the same time, the possibility of chemical interactions at the polymer–ITO interface is shown.

## 1. Introduction

Over the past few years, flexible electronics have been increasingly incorporated into our daily lives. The first smartphone with a flexible display was officially presented at the CES 2018 conference, and in 3 years almost all major players in this market have similar phone models in their lineup. At the same conference, LG also announced the first TV screen which can be rolled up. Today’s rapid development of flexible electronic devices is primarily due to significant progress in organic crystalline materials (OCMs) over the past 10 years. In contrast to the traditional complementary metal-oxide-semiconductor (CMOS) structures mounted on rigid substrates in modern electronics, OCMs, which offer flexibility, transparency, and cost and weight reduction, have shown great potential in optoelectronics applications including organic field-effect transistors, LEDs, organic photovoltaics, and various types of sensors [1,2].

One such class of promising OCMs for next-generation flexible electronics is ferroelectric polymers (a relatively new class of electroactive materials based on copolymers of PVDF and some nylons) [3]. At present, these materials are being actively studied and find application in various fields of science and technology: as piezo sensors [4,5], biocompatible prosthetic materials, nanogenerators/sensors [6,7,8], and adaptive optical system elements [8,9,10,11]. In contrast to traditional piezoelectrics based on oxide ceramics (PZT, quartz, lithium niobate, etc.), ferroelectric polymers have mechanical and technological flexibility and high impact toughness allowing their effective use as large area piezoelectric generators, high breakdown fields, transparency [12]. Application of transparent electrodes, such as indium tin oxide (ITO) [13] to these films makes it possible to create flexible transparent sound sources [14] or sub-screen sensors, such as fingerprint scanners [15], acousto-optical transducers (modulators) and other devices. The creation of structures based on flexible transparent ferroelectric polymers, which also have high pyroactivity [16] and exhibit high electrocaloric effect, will significantly increase the potential of this class of polymeric materials in flexible electronics devices; these structures can be obtained by adding nanoscale particles to the melted or dissolved PVDF matrix [17]. In contrast, layered structures also can be useful for various applications. For example, an aluminum electrode deposited on poly (ethylene-2,6 naphtalate) film was used as a substrate for three-layer hybrid material consisting of vacuum-deposited barium-strontium titanate coated on both sides with a spray-deposited PVDF-TrFE copolymer film [18]; this material has a significant piezoelectric response with low degradation after 340,000 mechanical bending cycles and can potentially be used in wearable energy collection devices. In opposition to layered structures, powder-filled PVDF films usually show significant light absorption. For example, filled with 15% BaTiO_3_ PVDF-PMMA composite, film with 0.012 cm thickness has an optical density of 0.7–1.0 in the visible light wavelength band [19].

Optical characteristics of PVDF-based composites also are being thoroughly investigated at the present time. Rare earth ions doped fibrous PVDF material, for example [20] can be used for converting near-infrared light to visible light. Owing to its high reflectance in the wide part of the IR optical band, PVDF copolymer fibers may be used as radiative cooling material [21].

This research is devoted to the study of some characteristics of hybrid materials obtained by depositing transparent ITO electrodes on a polarized PVDF film, with which, for example, acoustic waves can be generated when an alternating electric field is applied to it. The use of such a structure in flexible electronic devices is subject to a number of external factors. First of all, these are thermomechanical stresses associated with mechanical deformations and temperature effects during operation or during the deposition of conductive layers to the film. The paper presents a study of the structure by infrared spectroscopy and comparative results of tests of PVDF film before and after the deposition of ITO layers in uniaxial tension, its dynamic mechanical analysis, DSC, as well as measurements of transparency and piezoelectric properties of such a structure.

## 2. Materials and Methods

The objects of the study are samples of 50 µm-thick polarized PVDF-P00050 film produced by PolyK (State College, PA, USA) with a declared piezoelectric constant value of d_33_ over 30 pC/N. Some samples were coated on both sides of the film with 98 nm thick optically transparent electrically conductive ITO coating with a specific surface resistivity of 190 Ohm/□ (PVDF + ITO samples). The ITO was deposited by the reactive magnetron method from 2 metal (Sn and In) targets in an oxygen atmosphere on the QUADRA series quadrupole magnetron sputtering unit (NPF “Elan-Practic” Ltd., Russia). The magnetrons of the quadrupole system are evenly spaced around the carousel device, which ensures high plasma homogeneity during deposition and practically excludes the zones with low ionization degrees and low atom flux density of the deposited material [22]. In the deposition process, the polymer film is slowly heated for 10 min from 60 to 100 °C, stays at this temperature for 7 min in the process during the ITO layer deposition, and slowly cools down.

IR-spectra were recorded on a VERTEX 70v Fourier-transform IR-spectrometer using the attenuated total reflection (ATR) method using a Pike Glady ATR adapter with a diamond crystal in the 4000–400 cm^−1^ range and spectral resolution of 4 cm^−1^. Measured ATR spectra were corrected using OPUS 7 software to allow for the wavelength dependence of radiation penetration depth into the sample. The absorption spectra of the polymer films were measured on a TENSOR 37 Fourier-transform spectrometer with a spectral resolution of 2 cm^−1^. The structure of PVDF films was investigated by infrared spectroscopy in two stages. In the first stage, the role of high-temperature annealing (200 °C) on volumetric characteristics of the pure PVDF film was tested. In the second stage, PVDF + ITO samples were investigated before and after high-temperature annealing.

Thermal and physical-mechanical studies of PVDF film were performed by analogy with the studies of polylactic acid films, which we conducted earlier in [23].

Thermal properties of the film materials were determined using a differential scanning calorimeter NETZSCH DSC 204F1 Phoenix (Selb, Germany) in an inert atmosphere at a flow rate of argon 100 mL/min. Film samples (raw and coated) were heated in the temperature range from 20 °C to 200 °C at a rate of 10 °C/min. After the first heating cycle, the samples were kept at 200 °C for 5 min and cooled to room temperature, and then reheated to 200 °C at a rate of 10 °C/min to record the second DSC curve. These curves were used to determine the melting heat effect ($\Delta H_{m}$) and then to calculate the percentage of crystallinity ($\chi_{c}$) using the following expression:

(1) $$\chi_{c}\left(\%\right)=\frac{\Delta H_{m}}{\Delta H_{m}^{*}}\times 100\%$$

where $\Delta H_{m}^{*}$ = 105 (thermal melting effect for 100% crystalline PVDF [24])

The film samples were tested by the DMA method under tension on a NETZSCH DMA 242 E Artemis dynamic mechanical analyzer (Selb, Germany). The samples were coated and uncoated strips with a width of 5 mm and a length of the working part of 10 mm. Samples were cut out along the material orientation axis (direction 0) and transversely (direction 90). The cut specimens were tested under isothermal conditions at 30 °C The frequency of external load application was varied from 0.25 to 100 Hz.

**Figure 1:**
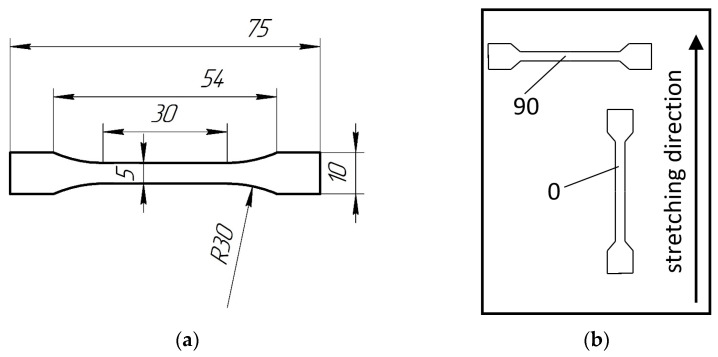
Dimensions of the film samples tested in tension (**a**) and the scheme of their cutting from the film (**b**).

Tensile mechanical characteristics of the films (original and coated) were determined on a Zwick Z100 testing machine (Ulm, Germany) at room temperature and a loading rate of 1 mm/min. The samples were also cut in two directions: 0 and 90 (Figure 1).

The given values (fracture strength σ_f_, maximum strength σ_m_, modulus of elasticity E, elongation at failure ɛ_f_, and elongation at maximum strength ɛ_m_) represent the arithmetic mean of the measurements for 5 samples for each series of specimens.

The piezoelectric coefficient d_33_ was measured according to the Berlincourt method [25] using a PKD3-2000 device (PolyK, State College, PA, USA) for 2 × 2 cm^2^ samples at 16 points of each sample. Measurements were performed at a calibrated force of 0.25 N on a frequency of 110 Hz.

The light transmission in the visible and IR wavelength range from 380 to 2600 nm was determined on a Shimadzu UV-3600i Plus spectrophotometer with a resolution of 1 nm at normal incidence of light on the sample using the integrating sphere.

Average light transmission and color rendering coefficients were calculated in accordance with European Standard EN 410:2011 Glass in building—Determination of luminous and solar characteristics of glazing.

## 3. Results

### 3.1. Structure of PVDF Films by DSC and IR Spectroscopy

**Figure 2:**
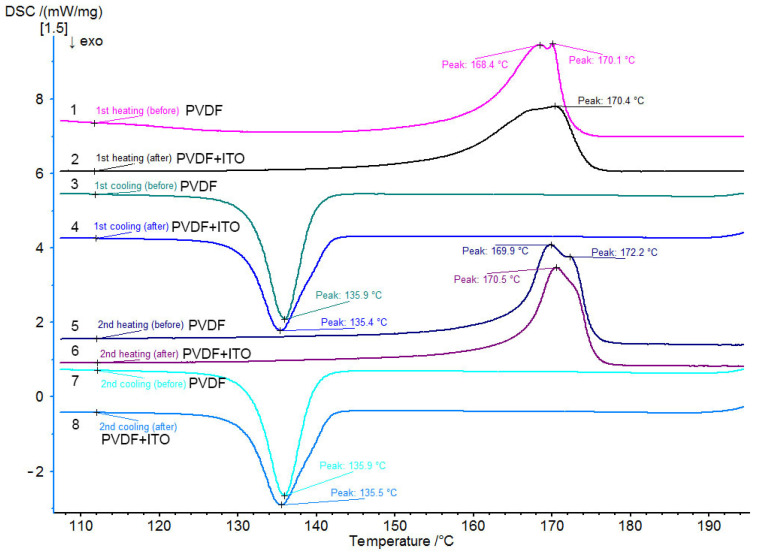
DSC curves for the original PVDF films without ITO (curves 1, 3, 5, 7) and with ITO (curves 2, 4, 6, 8).

Figure 2 shows the DSC curves obtained for the PVDF and PVDF + ITO films.

**Figure 3:**
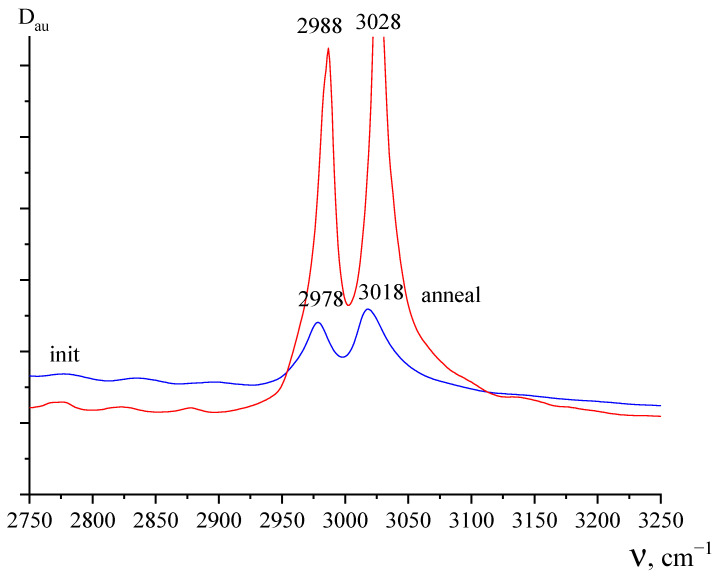
IR absorption spectrum in the range of 2750–3250 cm^−1^ for the original PVDF film (initial) and after heating to 200 °C (annealing).

**Figure 4:**
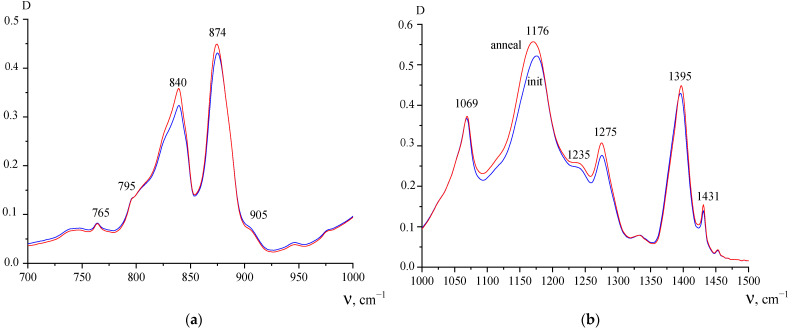
ATR IR absorption spectrum in the 700–1000 cm^−1^ (**a**) and 1000–1500 cm^−1^ (**b**) range for the original PVDF film (blue curve) and after heating to 200 °C (red curve).

The DSC curves obtained by heating the pure PVDF film show a double endothermic peak (T_m1_ and T_m2_, Figure 2) which corresponds to the characteristic melting temperature of the polymer. When the film is reheated, the double endothermic peak is also preserved, but the intensity of the peak changes (curve 5, Figure 2). The T_m1_ peak becomes more intense which indicates a change in the supramolecular structure. Based on the data obtained, we can assume that the supramolecular structure of PVDF is formed by two types of crystal structures. It is known [26,27,28] that PVDF macromolecules can have at least three types of crystal structures (α-, β- and γ- phases) depending on the macromolecule conformation. The double peak on the DSC curve describing PVDF melting indicates the simultaneous presence of α- and β-crystalline phases in the samples, which is confirmed by infrared spectroscopy data (Figure 3 and Figure 4). The double peak in the sample curve is preserved after deposition of ITO coating on its surface (Figure 2, curves 2, 6). It can be assumed that the coating conditions do not affect the supramolecular structure of PVDF film.

Thermograms of the cooling of PVDF samples (coated and uncoated) (Figure 2, curves 3, 4, 7, 8) are described by a single endothermic peak corresponding to PVDF crystallization. The peaks’ positions and shapes remain constant for PVDF and PVDF + ITO. It may be noted that the width of the crystallization peak in the PVDF films with ITO is noticeably wider than in the pure PVDF films.

The crystallization and melting temperatures as well as the thermal effects of melting and percent crystallinity are more clearly shown in Table 1.

The results of the study of the pure PVDF film by infrared spectroscopy before and after annealing are shown in Figure 3 and Figure 4. Figure 3 shows that doublet absorption spectrum bands (due to the symmetric antisymmetric component) in the region of valence vibrations of methyl groups noticeably shift toward higher frequencies after annealing. As follows from the DSC data, the melting peak is characterized by a doublet. One reason for this may be related to the polymorphism of PVDF crystals [1,2,3]. Since α-, β- and γ-phases have different chain conformations, they have their own absorption bands in the vibrational spectra. Figure 4a shows that in the initial state of the film, there is a large fraction of the polar β-phase observed, for which an intense absorption band of 840 cm^−1^ is responsible [1,2,3].

Since PVDF crystallization from the melt under normal conditions occurs in the α- phase, its conversion to the polar β- modification is usually performed by uniaxial stretching at low temperatures [1,2,3]. It is obvious that this also takes place for the studied film. Confirmation of this can be found in mechanical tests, where a large difference in the deformation behavior in two mutually perpendicular directions can be seen. However, along with the 840 cm^−1^ band, weak 765 and 795 cm^−1^ bands characteristic of the TGTG-chain conformation are observed in the spectrum, which indicate the presence of small amounts of α-phase [1,2,3]. It is the presence of the latter that can lead to the low-temperature endotherm. If this hypothesis is correct, then an increase in the α-phase fraction in the film should lead to an increase in its intensity. As can be seen from the DSC data, this is noted in the second heating cycle of the studied film. As follows from Figure 3, the absorption peaks in the film after annealing are shifted towards higher frequencies. As follows from the results of [3], this indicates an increase in the α-phase fraction during the annealing, which is confirmed by an increase in the low-temperature peak on the DSC during the second heating as compared to the first.

Other information can be obtained from Figure 4, namely the change in the microstructure of the film surface after annealing, since the micron-sized layer is investigated by ATR imaging. As follows from the above figures, the intensity of the 840 and 1275 cm^−1^ bands are practically unchanged after annealing. Since these bands are sensitive to the presence of long chain regions in the planar zigzag conformation [1,2,3], the reason for the observed change may be the pre-polarization of the film. As noted above, the initial film has a piezo effect.

**Figure 5:**
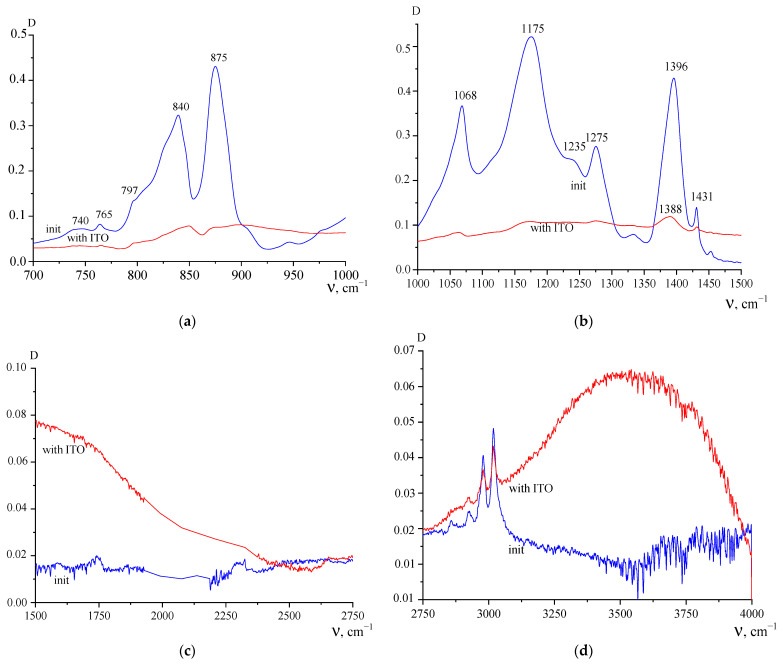
ATR IR absorption spectrum in the 700–1000 cm^−1^ (**a**), 1000–1500 cm^−1^ (**b**), 1500–2750 cm^−1^ (**c**) and 2750–4000 cm^−1^ (**d**) range for the original PVDF film (init) and with applied ITO electrodes.

Figure 5 shows that PVDF film with ITO coatings in the 700–1500 cm^−1^ range has strong absorption, as the bands characteristic of the polymer are almost absent in the coated film. This may be due to the fact that the thickness of the applied ITO turns out to be greater than the thickness of the probed layer. The possibility of chemical interactions at the PVDF–ITO interface can be judged from Figure 5c,d. This is indicated by the appearance of two wide maxima in the region of 1500 cm^−1^ and 3500 cm^−1^. They are visible only because, as can be seen, the polymer itself practically does not absorb in these spectral regions. Comparison with the spectrophotometer data gives, as can be observed, a qualitative correspondence.

**Figure 6:**
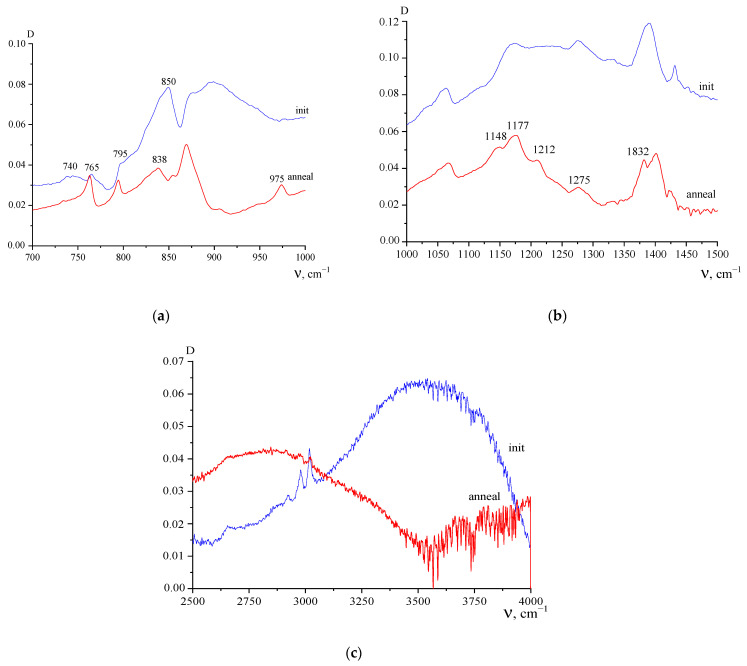
ATR IR absorption spectrum in the 700–1000 cm^−1^ (**a**), 1000–1500 cm^−1^ (**b**), and 2500–4000 cm^−1^ (**c**) range for PVDF and PVDF + ITO films with ITO before (init) and after annealing (aneal).

Thermal treatment of films with ITO electrodes (Figure 6) leads to an unusual effect: bands characteristic of the polymer appear in the spectral region after annealing. The reason for this phenomenon is not completely clear, but one reason may be related to thermally activated chemical reactions between ITO molecules and PVDF surface molecules. An indication of the possibility of such a reaction follows from Figure 6a. It shows that the wide ITO 3500 cm^−1^ absorption band after annealing is strongly shifted toward low frequencies.

**Figure 7:**
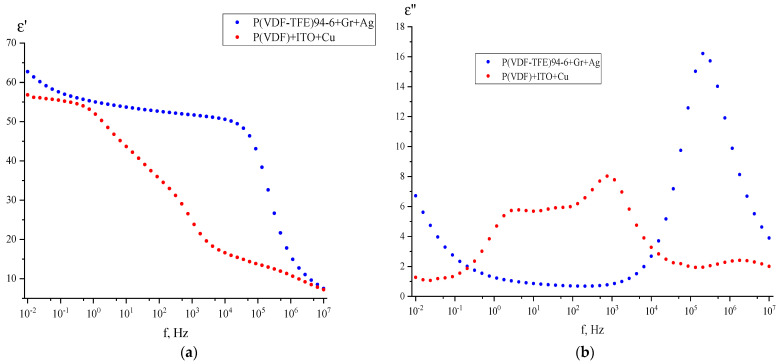
Frequency dependences of the real (**a**) and imaginary (**b**) components of the complex permittivity of PVDF with ITO.

The possibility of intensification of the noted chemical interactions at the polymer–ITO interface during annealing indirectly follows from the frequency dependences of the components of the complex permittivity of three-layer capacitors, metal-polymer-ITO-metal, which are shown in Figure 7a,b. For comparison, the curves for a similar copolymer with a conductive layer of graphene-containing material [29] are shown in the graph. It can be seen that in the case of the inorganic conductive coating, two new dispersion regions arise in the material at low frequencies.

### 3.2. Thermomechanical Test Results

**Figure 8:**
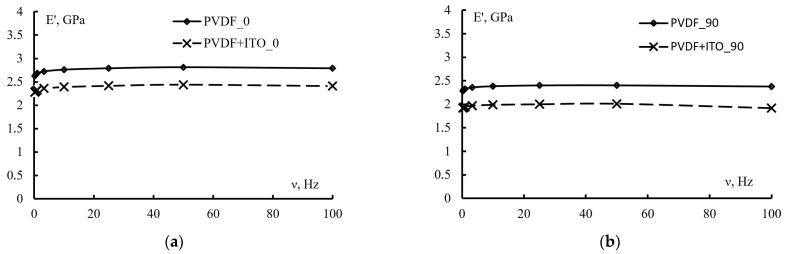
Variation of the elastic modulus E′ in direction 0 (curve 1) and in direction 90 (curve 2) with increasing frequency v for the original and coated PVDF films in directions 0 (**a**) and 90 (**b**).

The change in elastic modulus of PVDF films with increasing frequency of load application is shown in Figure 8. It can be seen that in all cases, the elastic modulus changes little in the frequency range of 0.25 to 100 Hz. At the same time, the change in E′ values is about 5%. It should be noted that the curves describing the change in elastic modulus of PVDF and PVDF + ITO films with frequency are at a different level. The difference between the modulus E′ values is about 15%.

Table 2 (E’ at a fixed frequency (50 Hz)) shows that elastic modulus differs by about 15% for the specimens tested in and across the orientational tensile direction. The coating decreased the elastic modulus by about 15%. At the same time, the difference in the E′ values for directions 0 and 90 remained at the same level.

**Figure 9:**
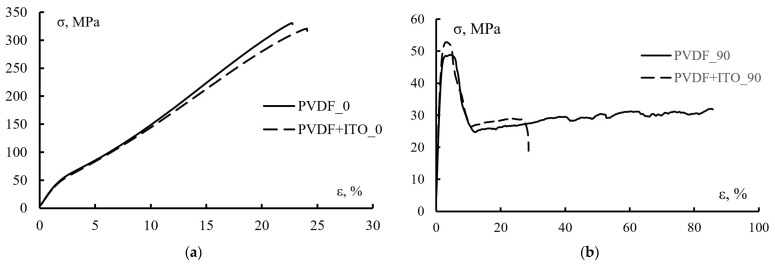
Typical load diagrams for the original and coated PVDF films in directions 0 (**a**) and 90 (**b**).

Figure 9 shows typical load diagrams of coated and uncoated PVDF film. In direction 0, the studied samples (coated and uncoated) have the same load diagrams. In both cases, stresses grow elastically at small deformations. After reaching a strain of 1.5%, the specimens begin to deform irreversibly. At about 3% strain (stress of about 50 MPa), a small yield point is observed. With further increase in strain, stresses increase until the materials fracture.

The tensile diagrams of the samples cut in the 90 direction are significantly different from the diagrams described above. As in the previous case, the treated and untreated films deform elastically until the relative elongation reaches 1.5%. With further increase in strain, a maximum of stresses corresponding to the temporary strength of the material is observed. It should be noted that the maximum strength value for specimens cut in the 90 direction approximately corresponds to the “yield strength” of specimens cut in the 0 direction. Furthermore, there is a decrease in stresses to the level of 20–25 MPa and an increase in elongation up to the point of specimen failure. It should be noted that the coating deposition influenced the film deformability in direction 90.

The change in the elastic-strength properties during coating is shown more clearly in Table 3.

It can be seen that the maximum strength σ_m_ in direction 0 for the materials with and without treatment is almost the same, but differs significantly for different orientation directions (almost six times greater in direction 0). This difference in maximum strength values indicates a high degree of film orientation. Tensile strength changes similarly. The high degree of orientation of the film is evidenced by its deformation corresponding to the maximum strength and fracture strength. Thus, the fracture strain ɛ_f_ for the pure PVDF film differs almost 5 times depending on its orientation (22.4% and 100% respectively for orientation 0 and 90). Additionally, if coating deposition practically does not change ɛ_f_ in the case of orientation 0, then in the direction of 90 ɛ_f_ decreases by up to 20%. Such a sharp decrease in the deformability in the direction 90 is due to the ITO deposition.

The result of determining the elastic modulus was somewhat unexpected, which for the untreated PVDF film in both directions left about 3.1 GPa, and after coating deposition it decreased to 2.87 GPa in the 0 direction and increased to 3.47 GPa in the 90 direction.

It should be noted that the modulus determined by the DMA method differs markedly from the elastic modulus measured under quasi-static loading conditions. Thus, the elastic moduli in directions 0 and 90 differ by 10% and 20%, respectively.

### 3.3. Optical and Piezoelectric Properties

The value of the piezoelectric coefficient d_33_ for the initial films is 28 ± 3 pC/N (corresponds to the value declared by the manufacturer), and after the deposition of ITO, respectively, 25 ± 6 pC/N. It can be seen that the average value of the piezoelectric modulus d_33_ after ITO application slightly decreases, although the error increases.

**Figure 10:**
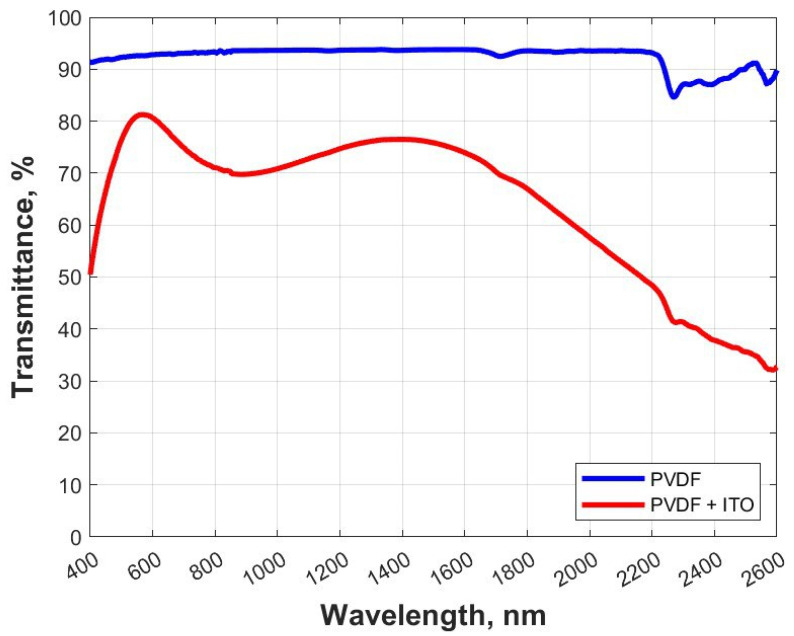
Transmittance for PVDF and PVDF + ITO films.

The dependences of the transmittance coefficients of the experimental samples on the wavelength of the visible and IR ranges are shown in Figure 10.

The determined values of light transmission coefficients, the average value of transmittance coefficients in the IR wavelength range, and color rendering coefficients of experimental samples are presented in Table 4.

The transparency of the ITO-coated samples decreased by 13% in the visible wavelength range and by almost 30% in the IR wavelength range from 780 to 2600 nm. The color rendering of the ITO-coated sample even improved slightly compared to the original sample.

## 4. Discussion

As expected, the results of the studies revealed that the viscoelastic behavior of the films under tension along and across the orientation is described by different loading diagrams because the initial PVDF film was uniaxial stretched. Therefore, the strength of uncoated and coated PVDF films differs by an order of magnitude depending on the test direction.

PVDF is characterized by two melting temperatures, which indicates the presence of two types of crystal structures (α- and β- phases) in the polymer supramolecular structure. IR spectroscopy indicates the predominance of the β-phases, which is confirmed by the high piezoelectric constant of the initial PVDF film.

Heating the PVDF film to 200 °C (above the melting point) leads to a slight increase in the α-, which is confirmed by DSC (increasing T_m1_ peak intensity) and IR spectroscopy. Despite the fact that PVDF crystallization usually takes place in the α-phase, the β-phase is still present after heating the films above the melting temperature, which can be explained by the preliminary polarization of the film. This indicates that the polarized PVDF film retains its ferroelectric properties even after heating to the melting temperature.

The temperature-time mode of ITO deposition has little effect on the structure, thermophysical and mechanical properties of the polymer. The maximum strength σ_m_ in direction 0 and the shape of the DSC curves during heating for the samples with and without ITO are almost the same. The piezoelectric constant d_33_ also changed slightly (from 28 to 25 pC/N). This allows us to conclude that the ITO deposition process had practically no effect on the internal structure of the polymer material.

At the same time, the results of the annealing of PVDF films with ITO indicate the possibility of chemical interactions at the polymer–ITO interface that is indirectly confirmed by the frequency dependences of the components of the complex permittivity of three-layer metal-polymer-ITO-metal capacitors.

The possibility of such interactions is confirmed by the broadening of the crystallization peaks on the DSC curve after ITO deposition. The chemical interaction between ITO and the surface layer of the polymer can interfere with the crystallization process during cooling, and the transition of the melt into a crystal will occur in a wider temperature range. The slight decrease in crystallinity from 48% to 44% can be explained by structural disturbance in the polymer surface layer due to the interaction of ITO with polymer chains.

This interaction can also affect the mechanical properties of the ITO-PVDF-ITO structure. This can explain why the deposition of ITO films affected the film stretch in the 90 direction decreasing the film strain by a factor of 5 times or the unexpected elastic modulus results noted above.

## 5. Conclusions

In this paper, we demonstrated flexible transparent film ITO-PVDF-ITO with transparency of 79.6%, a color rendering index of 84, and a piezoelectric coefficient d_33_ 25 ± 6 pC/N and investigated the effect of the ITO deposition technological process on the properties of the PVDF film. The manifestation of memory effects was noted for the initial film. It is shown by the fact that the characteristics of conformationally sensitive absorption bands of the film change weakly when the temperature rises even above the melting point. Hypotheses for this phenomenon are proposed. Infrared spectroscopy in the ATR variant demonstrates chemical interactions of ITO molecules with the polymer surface layer where an increased content of defects of both chemical and physical nature is observed for PVDF films with an applied ITO layer. It can be concluded that the deposition process has almost no effect on the mechanical, structural, and piezoelectric properties of initial PVDF film taking into account the operation of such structures in the elastic region, decreasing the transparency of pure PVDF film by 14% in the visible range. The discovered possible chemical interactions between ITO and PVDF can provide good adhesion of ITO layers to the polymer and expand the application potential of such films.

## Figures and Tables

**Table 1:** Melting and crystallization temperatures, melting thermal effects, and percent crystallinity for PVDF films with and without ITO.

Sample	T_m1_, °C	T_m2_, °C	T_cr_, °C	ΔH_m_, J/g	χ_c_, %
PVDF, heating 1	168	170	136	50	48
PVDF, heating 2	170	172	136		
PVDF + ITO heating 1	168	170	135	46	44
PVDF + ITO, heating 2	170	172	136		

**Table 2:** Elastic moduli E’ of coated and uncoated PVDF films at a load frequency of 50 Hz.

Cutting Direction	E’, GPa (PVDF)	E’, GPa (PVDF + ITO)
0	2.8	2.4
90	2.4	2.0

**Table 3:** Elastic strength properties of PVDF film.

Sample	E, GPa	σ_m_, MPa	ɛ_m_, %	σ_p_, MPa	ɛ_f_, %
PVDF_0	3.10 ± 0.10	334 ± 15	22.5 ± 1	330 ± 18	22.4 ± 10.0
PVDF_90	3.11 ± 0.13	49 ± 2	4.3 ± 0.3	29 ± 3	100.5 ± 42.1
PVDF + ITO_0	2.87 ± 0.06	305 ± 7	24.0 ± 0.1	289 ± 15	25.7 ± 2.4
PVDF + ITO_90	3.47 ± 0.11	52 ± 2	3.1 ± 0.1	17 ± 5	19.4 ± 7.4

**Table 4:** Optical properties of experimental samples.

Sample	Average Transmittance in the Visible Range, %	Average Transmittance in the IR Range from 780 to 2600 nm, %	Color Rendering Index
PVDF	92.6	92.4	81.0
PVDF_ITO	79.6	62.0	84.6

## Data Availability

The data presented in this study are available on request from the corresponding author.
